# A tissue‐resolved, network‐based transcriptomic framework for abiotic stress responses in sorghum

**DOI:** 10.1111/tpj.70834

**Published:** 2026-03-29

**Authors:** Dae Kwan Ko, Federica Brandizzi

**Affiliations:** ^1^ MSU‐DOE Plant Research Lab Michigan State University East Lansing Michigan 48824 USA; ^2^ Department of Plant Biology Michigan State University East Lansing Michigan 48824 USA; ^3^ Great Lakes Bioenergy Research Center Michigan State University East Lansing Michigan 48824 USA

**Keywords:** gene regulatory network, co‐expression network, abiotic stress, bioenergy crops, tissue specificity, spatiotemporal dynamics, transcriptomics

## Abstract

Developing climate‐resilient crops requires a detailed understanding of stress‐induced gene expression dynamics, as maladaptive responses can compromise their productivity and survival. Sorghum, a globally important cereal with exceptional tolerance to multiple abiotic stresses, provides a powerful system for investigating these dynamics. However, how stress type, tissue specificity, and temporal progression jointly shape transcriptomic responses in crops remains poorly understood. Here, we present a comparative, time‐resolved transcriptomic atlas of sorghum responses to drought, heat, and salinity stress across shoot and root tissues. Integrative analyses revealed that tissue specificity is the dominant determinant of abiotic stress‐induced gene reprogramming across all three stresses. Building on these global comparisons, we focused on heat stress, as it elicited the most coherent and pronounced transcriptional and regulatory responses, enabling deeper network‐level interrogation. Co‐expression network analysis identified tissue‐specific modules enriched for phytohormone‐responsive genes, while gene regulatory network (GRN) mapping and cistrome analyses uncovered transcription factors (TFs) controlling key hub genes within these modules. Together, this study provides a foundational transcriptomic and network‐based resource for dissecting the regulatory architecture of abiotic stress responses in sorghum and offers prioritized candidates for future functional validation and engineering of climate‐resilient crops.

## Significance Statement

Understanding how crops coordinate gene expression across tissues and time under environmental stress is a major challenge for improving climate resilience. This study provides a comprehensive, network‐based transcriptomic resource that reveals tissue specificity as the dominant driver of abiotic stress responses in sorghum, offering a valuable framework and prioritized regulatory candidates for future crop improvement.

## INTRODUCTION

Over the course of 100 million years of evolution (Leslie et al., 2021), flowering plants have adapted to diverse environments by evolving intricate cellular, molecular, and physiological mechanisms (Anderson et al., 2011; Franks & Hoffmann, 2012; Parmesan, 2006). However, the unprecedented rate of climate change now poses severe threats to the global ecosystem and ultimately food security, with the frequency and intensity of environmental extremes projected to increase (Fedoroff et al., 2010; Sala et al., 2000; Sillmann et al., 2013). Among these extremes, heat stress, driven by increasingly frequent and intense heatwaves, has emerged as a major constraint on crop growth, productivity, and quality worldwide (Mazdiyasni & AghaKouchak, 2015; Zhao et al., 2017). Importantly, heat stress rarely occurs in isolation (Zhang et al., 2022). Abiotic stressors, such as heat, drought, and salinity are tightly interconnected: elevated temperatures accelerate evapotranspiration, intensifying drought stress (Lamers et al., 2020; Zhang et al., 2022), while reduced water availability can promote salt accumulation in soil, exacerbating salinity stress and further compromising crop yields (Melino & Tester, 2023; Munns & Gilliham, 2015). These concurrent stresses can significantly reduce crop yields, highlighting the urgent need for a deeper understanding of plant stress responses.

To survive and thrive in extreme conditions, plants undergo extensive transcriptional reprogramming for essential cellular and metabolic pathways through the interplay of *cis*‐regulatory elements (CREs) and TFs (Cerda & Alvarez, 2024; Kidokoro et al., 2022; Melino & Tester, 2023; Zhang et al., 2022). Effective stress responses are shaped by three key determinants: stress type, temporal dynamics, and tissue specificity (Alvarez et al., 2021; Swift et al., 2022). Stress‐specific transcriptomic responses co‐exist with shared regulatory mechanisms, enabling flexible and context‐dependent responses (Shaar‐Moshe et al., 2017; Zhang et al., 2022). Moreover, transcriptional responses are often multiphasic, involving early pro‐survival and later adaptive or trade‐off associated pathways (Song et al., 2016; Srivastava et al., 2018). These temporal dynamics intersect with spatial constraints, as stress perception and response differ markedly across tissues (Pérez‐Díaz et al., 2014; Yu et al., 2023). For example, heat stress primarily affects above‐ground tissues before systemic signaling modulates root responses (Gilroy et al., 2014; Zandalinas et al., 2020), while salt primarily targets below‐ground tissues and subsequently alters shoot physiology (van Zelm et al., 2020). Together, these determinants organize gene regulatory networks (GRNs) (Alvarez et al., 2021; Bonneau, 2008; Ko & Brandizzi, 2020; Swift et al., 2022), which remain less well characterized in crops than in model species (Ko & Brandizzi, 2020; Lavarenne et al., 2018; Springer et al., 2019).

Sorghum (*Sorghum bicolor*), the fifth most important cereal crop globally and a major bioenergy feedstock (Mullet et al., 2014), exhibits innate tolerance to drought, heat, and salinity (Djanaguiraman et al., 2020; Mace et al., 2013), making it an excellent model for dissecting abiotic stress responses in crops. Previous studies have documented extensive transcriptional reprogramming in sorghum under drought (Varoquaux et al., 2019), heat (Johnson et al., 2014), and salinity (Sui et al., 2015). However, these studies have typically focused on individual stresses, single tissues, or limited time points, restricting direct comparisons of how stress type, tissue specificity, and temporal dynamics collectively shape regulatory responses. For instance, drought affects nearly half of the sorghum transcriptome (Varoquaux et al., 2019), whereas heat and salinity preferentially induce distinct transcriptomic shifts in metabolic and hormonal pathways (Johnson et al., 2014; Sui et al., 2015). A unified comparative framework is therefore required to elucidate both shared and stress‐specific regulatory strategies.

In this study, we employed a network‐enabled, integrative transcriptomic approach to dissect the interplay among stress type, tissue specificity, and temporal dynamics in sorghum responses to drought, heat, and salinity. By generating a time‐course transcriptome dataset across shoot and root tissues, we first established global, cross‐stress patterns and identified tissue specificity as the dominant determinant of abiotic stress‐induced gene expression. Building on these comparative analyses, we then used heat stress for deeper network‐ and mechanism‐level interrogation, as it produced the most consistent and pronounced transcriptional and regulatory reprogramming across tissues. Through co‐expression and GRN analyses, we identified tissue‐specific modules enriched for hormone‐responsive genes, along with key hub genes and upstream TFs. This framework provides mechanistic insight into stress‐responsive regulatory hierarchies and offers a foundation for engineering climate‐resilient crops.

## RESULTS

### A comprehensive atlas of abiotic stress‐inducible gene expression changes in sorghum

To assess the impact of recent global warming on sorghum production, we analyzed long‐term trends in temperature and sorghum yield in the United States from 1961 to 2022, along with their relationship (Figure S1a; Table S1). Although sorghum production has shown moderate increases from 1961 to 2022, largely due to robust breeding programs and improved agricultural practices, such as irrigation, pesticides, and fertilizers (Hao et al., 2021), the recent rise in temperatures has had a devastating impact. This is evidenced by the negative correlation between sorghum yield and rising temperatures, particularly from 2001 to 2022 (Figure S1b). These findings underscore the pressing need to elucidate the molecular mechanisms underlying sorghum's resilience to environmental stressors, which are being exacerbated by climate change projections (Mazdiyasni & AghaKouchak, 2015; Sala et al., 2000). We hypothesized that sorghum's resilience to abiotic stresses is governed by cumulative modes of gene action, coordinated by master TFs within a regulatory hierarchy. To test this, we aimed to create a comprehensive atlas of gene reprogramming in sorghum under drought, heat, and salinity stresses.

We performed RNA‐seq on shoot and root at four key time points of stress application (6 h, Early [E]; 24 h, Middle 1 [M1]; 48 h, Middle 2 [M2]; 72 h, Late [L]) selected based on their significance in prior studies (Coolen et al., 2016; Dugas et al., 2011; Ko & Brandizzi, 2022) and their relevance to well‐characterized phases of stress‐induced transcriptional reprogramming preceding metabolic and physiological changes (Buchanan et al., 2005; Chiluwal et al., 2020; Fontanet‐Manzaneque et al., 2024; Johnson et al., 2014) (Figure S2a; Data S1). Sorghum seedlings were subjected to drought, heat (45°C), and salinity stresses (200 mM NaCl) beginning at 13 days after planting (DAP), except for drought, where water was withheld starting at 8 DAP. To validate the effectiveness of drought stress in our system, we measured soil water content at three critical time points: 8 DAP (onset of water withholding), 13 DAP (collection of 6 h samples), and 16 DAP (collection of 72 h samples). Soil water content decreased by 62% at 13 DAP relative to 9 DAP and by an additional 33% at 16 DAP relative to 13 DAP (Figure S2b), confirming that drought stress was effectively applied. This experimental design allowed for temporal comparisons of transcriptomic changes across stressors despite the inherent differences in stress initiation and progression (i.e., 4 h of drought stress is not equivalent to 4 h of heat). Parallel RNA‐seq profiling was also performed on unstressed control samples for all conditions (Figure S2a). This design allowed stress‐induced gene expression to be compared directly to time‐matched controls, eliminating potential diurnal effects.

**Figure 1:**
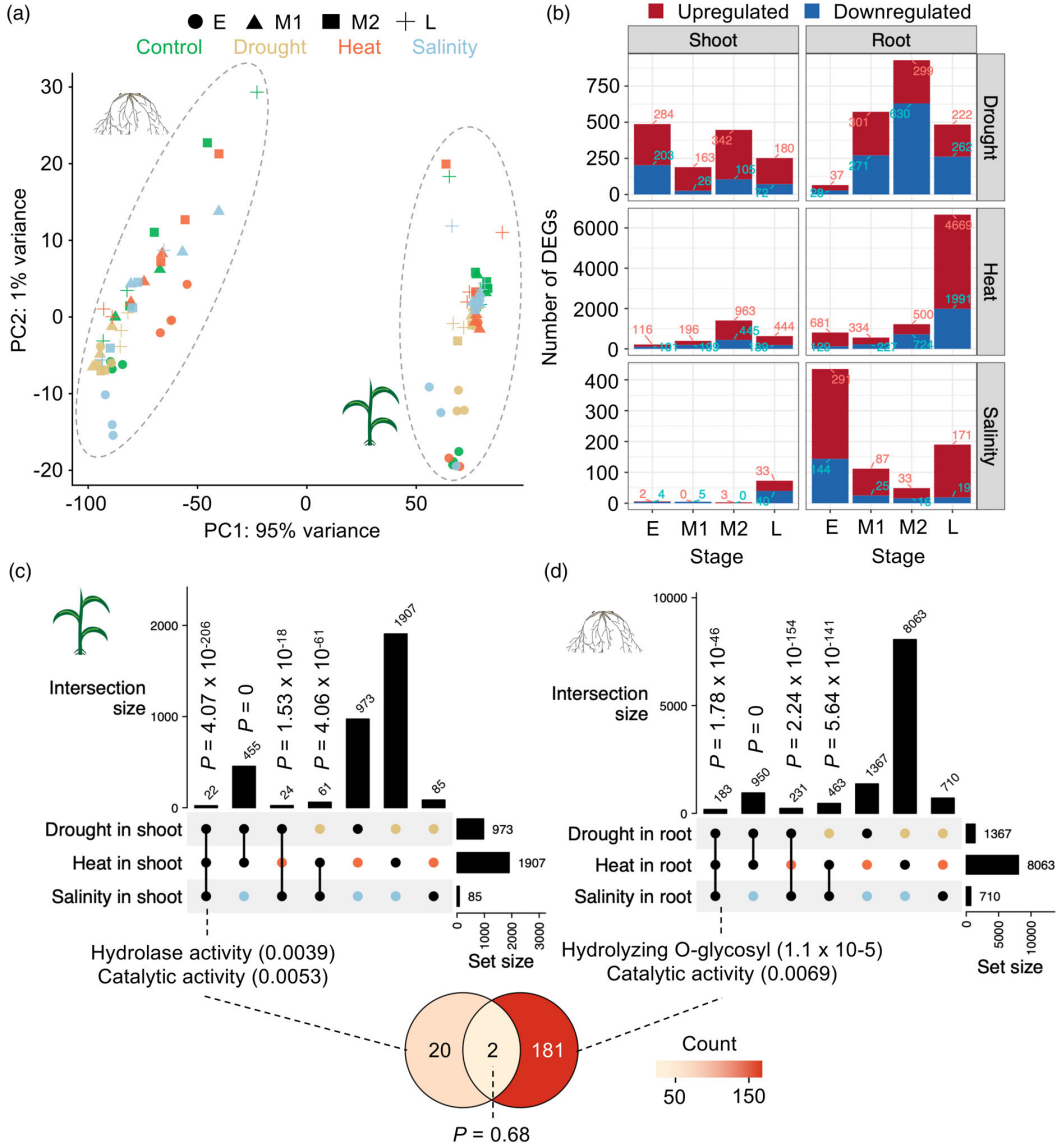
Interwinding of spatial, temporal, and stress‐specific transcriptomic dynamics in sorghum. (a) PCA of transcriptomic variation across all samples. PCA of variance‐stabilized gene expression values (vst() from DESeq2) was performed using all RNA‐seq samples to assess the dominant sources of transcriptomic variation. Each point represents an individual biological replicate. Samples are colored by stress condition (Control, Drought, Heat, and Salinity) and shaped by tissue type. The percentages of variance explained by the first two principal components (PC1 and PC2) are indicated on the axes. (b) Number of DEGs in each tissue at each time point under different stress conditions. Numbers are shown within the graphs. (c, d) Upset plots displaying the intersection among DEGs obtained under drought, heat, or salinity in shoots and roots. The top‐ranked GO terms for DEGs identified in all three stress conditions are shown. Venn diagram illustrating the minimal overlap between DEGs found under all conditions in shoots and roots.

Because our study systematically compared three key factors (tissue, temporal, and stress specificity) within a unified analytical framework, we sought to identify the most dominant factor driving transcriptomic reprogramming by performing principal component analysis (PCA) using the full RNA‐seq dataset encompassing all samples, which exhibited high reproducibility across biological replicates based on pairwise correlation analysis (Figure S2c). Our analysis revealed tissue type as the dominant factor driving transcriptomic responses, with clear distinctions between shoot and root samples (Figure 1a). Following the tissue‐specific clustering, samples grouped strongly by time point, with minimal clustering by stress type. This observation aligns with prior findings that drought, heat, and salinity stresses elicit overlapping signaling pathways in plants (Shaar‐Moshe et al., 2017; Uygun et al., 2019). Furthermore, we performed PCA separately for root and shoot samples to capture differences among stress treatments and time points more effectively (Figure S2d,e). In shoots, heat‐stressed samples clustered closely together. In contrast, root samples were more widely distributed, primarily according to the type of stress. Notably, the 72 h heat stress data in roots formed a distinct cluster, reflecting a pronounced transcriptional response. These results confirm that tissue type is the primary driver of transcriptomic variation, while time and stress type contribute additional, intertwined effects.

Next, we identified DEGs by comparing expression for each tissue type, time point, and stress conditions against controls (adjusted *P* < 0.05 and absolute Log_2_FC >1), revealing a total of 2447 DEGs in shoots and 8967 DEGs in roots across the different stressors (973 and 1367 for drought; 1907 and 8063 for heat; 85 and 710 for salinity in shoots and roots, respectively) (Figure 1b; Figure S3; Data S4 and S5). DEG patterns suggest a broader impact of drought stress across time points and tissues, with bimodal peaks in shoots and a single peak in roots, contrasting with heat stress, which exhibited a single peak in shoots and bimodal peaks in roots until the L phase, when DEG numbers increased exclusively in roots in line with the fact that heat has the most profound effect on the plant transcriptome. Salinity stress had an early‐phase effect primarily in roots. Notably, DEGs significantly overlapped among stresses within each tissue (Figure S4), in line with previous reports of the interconnectedness of abiotic stress responses at molecular and physiological levels (Shaar‐Moshe et al., 2017; Zandalinas et al., 2020, 2021). For example, 22 and 183 DEGs were common to all three stressors in shoots (*P* = 4.07 × 10^−206^) and roots (*P* = 1.78 × 10^−46^), respectively, with identical gene ontology (GO) terms (hydrolase and catalytic activities) ranking highest (Figure 1c,d; Data S3), supporting previous findings that abiotic stress‐induced metabolic enzymes with these activities are crucial for plant stress resilience (Hou et al., 2017; Monroe et al., 2014; Zanella et al., 2016). Despite these shared characteristics, the overlap between these DEGs in shoots and roots was minimal (2 out of 203), underscoring the tissue‐specific nature of stress‐induced transcriptomic changes despite an association with identical biological pathways.

To assess the consistency of our results with previous studies, acknowledging that abiotic stress assays often vary due to differences in experimental conditions and growth settings, we compared our data to Johnson et al. (2014), who reported heat‐ and drought‐responsive transcriptomes in sorghum (R16 variety) shoots (Figure S5a,b). Intersection analysis revealed significant overlap between our drought‐ and heat‐responsive DEGs and those reported by Johnson et al. (2014) (*P* = 1.59 × 10^−3^ for drought; *P* = 2.30 × 10^−75^ for heat), despite differences in growth conditions, genotypes (BTx623 versus R16), and technologies (RNA‐seq versus microarray). We also compared our DEGs with those reported by Varoquaux et al. (2019) under drought stress and observed similarly significant overlaps (*P* = 3.71 × 10^−101^ in shoots; *P* = 4.25 × 10^−68^ in roots) (Figure S5c,d). These results demonstrate the robustness and reproducibility of our findings, despite differences in experimental conditions and genotypes across studies. To validate our RNA‐seq results, we also performed quantitative real‐time PCR (qRT‐PCR) on nine stress marker genes (three per stress type) (Bi & Wang, 2022; Buchanan et al., 2005; Johnson et al., 2014) that were included in our DEG lists, generating a total of 69 data points. The qRT‐PCR data showed strong pairwise correlation with the corresponding RNA‐seq log_2_‐transformed fold‐change values in both shoots (*R* = 0.90, *P* = 6.50 × 10^−14^) and roots (*R* = 0.88, *P* = 2.50 × 10^−11^) (Figure S5e). Taken together, the extensive number of DEGs, their distinct patterns across samples, the strong concordance with previously identified stress‐responsive genes, and the high reproducibility of our dataset collectively underscore the robustness and depth of our transcriptomic analysis. This comprehensive transcriptional landscape provides a valuable resource for exploring abiotic stress‐responsive gene expression in sorghum and related grass species.

### Co‐expression network analysis of abiotic stress‐inducible transcriptomes

To better understand the gene programming patterns triggered by each stressor, we performed co‐expression network modeling analysis on the 2447 and 8967 DEGs identified at least at one time point in shoot and root tissues, respectively (Figure S6). Using WGCNA (Langfelder & Horvath, 2008) across all samples, representing the wide range of time points for each stress condition (i.e., drought E, M1, M2, L; heat E, M1, M2, M3; salinity E, M1, M2, L), we identified modules of co‐expressed genes (Figure S7; Data S6 and S7). Each module is summarized by a representative eigengene, the first principal component of the expression matrix, that captures the dominant expression pattern across conditions. WGCNA also quantifies the strength of co‐expression relationships within each module. In total, we identified 13 co‐expression modules in shoots and 17 in roots, each displaying distinct expression patterns across time points and stress types. Many of these modules are enriched for significant but distinct biological processes, such as cell redox homeostasis in the pink module (shoots) and cell wall biogenesis in the red module (roots) (Data S8 and S9). For instance, in shoots, blue module (213 genes) was strongly induced by drought stress at M2 phase, with transmembrane transport as the top GO term. Similarly, lightblue module (226 genes) exhibited M1‐specific induction by both drought and heat stresses, with protein phosphorylation as the top‐ranked GO term. In roots, the red module (4632 genes) showed a significant induction by heat stress during L phase, with cell wall biogenesis as the top GO term, while dark gray module (104 genes) displayed a late induction by both heat and salinity stresses. These findings underscore the ability of our approach to capture a wide array of co‐expression modules based on transcriptomic dynamics, providing a foundation for exploring the regulatory mechanisms and functional implications for stress resilience in sorghum.

**Figure 2:**
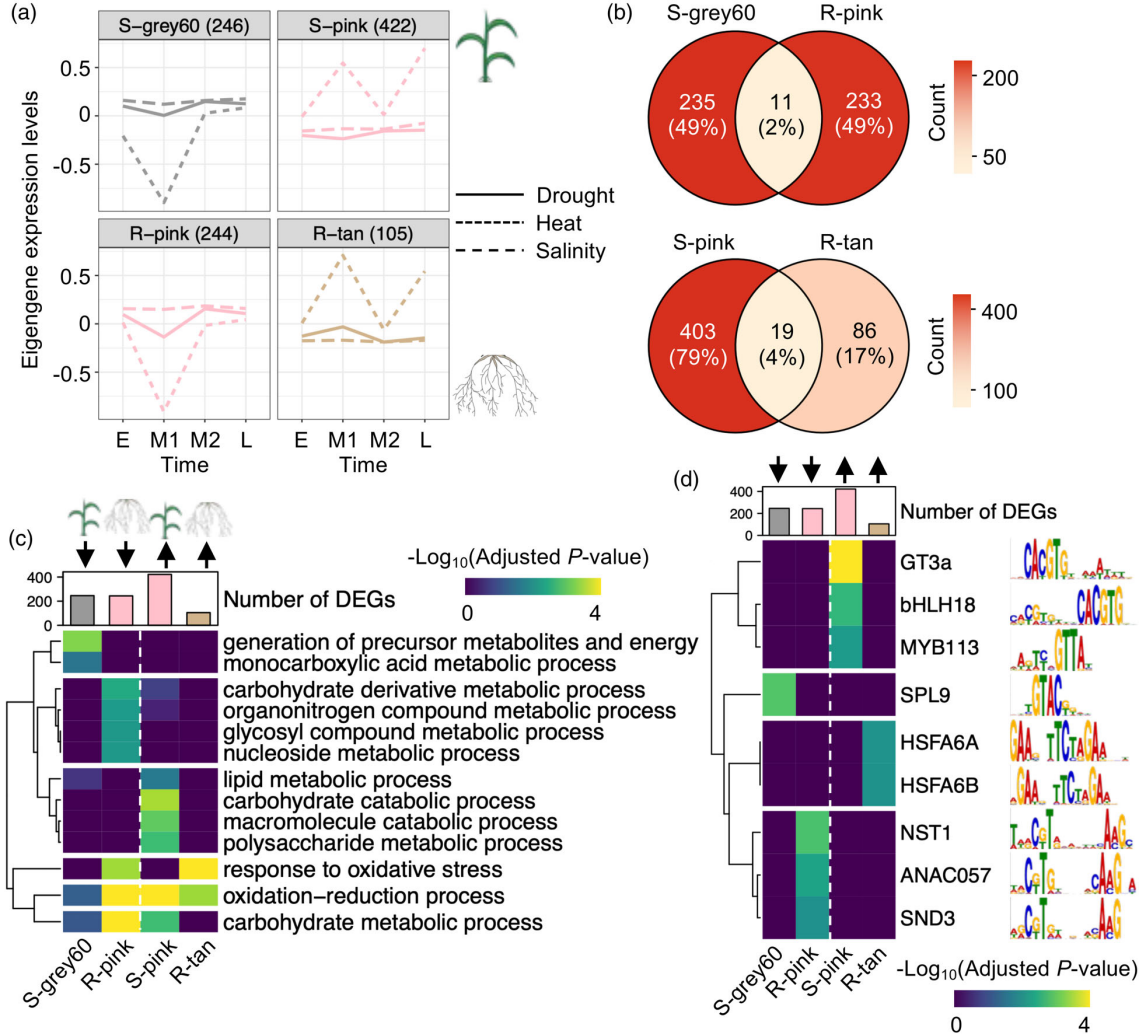
Co‐expression modules with identical expression patterns in shoot and root tissues comprise distinct genes. (a) Selected co‐expression modules generated by WGCNA. All DEGs in each tissue type (2447 in shoots and 8967 in roots) were subjected to WGCNA. Each graph displays the expression of the module eigengene, representing the average expression profile of the respective co‐expression module. The *x*‐axis indicates time points. Modules are named according to their WGCNA‐assigned color. The number of genes in each module is indicated in parentheses. (b) Intersection between the two modules with identical expression patterns. The number of DEGs in each segment is shown, along with the percentage in parentheses. The heatmap shows the count of DEGs. (c) Heatmap visualizing GO term enrichment in the heat‐induced downregulation (S‐gray60 and R‐pink) and upregulation (S‐pink and R‐tan) modules. The respective module is indicated by the color bar above the heatmap. Complete lists of GO terms enriched are provided in Data S8 and S9. (d) Heatmap exhibiting enrichment of TF DNA‐binding motifs on the 1‐kb promoters of genes that belong to the heat‐induced downregulation (S‐gray60 and R‐pink) and upregulation (S‐pink and R‐tan) modules. The corresponding TF family for each motif is displayed to the right of each row. The TF DNA‐binding motifs are depicted as sequence logos.

Next, to investigate tissue‐specific gene reprogramming under abiotic stress, we focused on identifying co‐expression modules that exhibit consistent expression patterns between shoots and roots. Comparing these modules offers insights into how different tissues either coordinate or diverge in their transcriptional responses to environmental stress. We identified two such module pairs that exhibited highly coherent, heat‐responsive expression dynamics across tissues: the gray60 module in shoots (S‐gray60) corresponded to the pink module in roots (R‐pink), and the pink module in shoots (S‐pink) matched with the tan module in roots (R‐tan) (Figure 2a). S‐gray60 and R‐pink exhibited heat‐specific expression suppression during the Middle phase. In contrast, S‐pink and R‐tan showed increased eigengene expression under heat stress during both the M1 and L phases, and they displayed a modest opposing trend between M1 and M2 under drought stress, although the magnitude of this change was small, limiting its biological interpretation. Despite the similar expression dynamics, the overlap of genes between shoot and root modules was minimal (only 2% between S‐gray60 and R‐pink, and 4% between S‐pink and R‐tan), highlighting the strong tissue specificity observed earlier (Figure 2b). Consistent with this, GO analysis revealed that each module was enriched for distinct biological processes, such as energy metabolism (generation of precursor metabolites and energy; monocarboxylic acid metabolic process) in S‐gray60 and oxidative stress (response to oxidative stress; oxidation–reduction process) in R‐pink (Figure 2c). Furthermore, *de novo* motif analysis on 1‐kb promoters of genes that belong to each module uncovered distinct sets of TF‐binding motifs enriched in the promoter regions of each module, suggesting that these expression patterns are independently regulated by unique TFs and their associated CREs (Figure 2d).

Collectively, these results indicate that co‐expression modules with similar stress‐responsive expression profiles in shoots and roots consist of largely distinct gene sets, functional pathways, and regulatory architectures. Based on their comparable gene counts, distinct GO enrichments, and robust, heat‐specific eigengene suppression shared across tissues, we selected S‐gray60 and R‐pink as a representative module pair for detailed network analysis. This analysis examines heat stress as a case study while maintaining the broader comparative context of all three stresses.

### Characterizing S‐gray60 and R‐pink modules at the network level

Because heat stress elicited the clearest and most synchronized transcriptional suppression in these modules, it provided a tractable context for dissecting tissue‐specific regulatory architectures. Under stress conditions, plants reallocate energy and resources from growth toward defense mechanisms (Cramer et al., 2011). This trade‐off is mediated by complex signaling networks integrating developmental and environmental cues, including key signal transduction pathways such as phytohormone signaling, redox dynamics, and lipid and metabolite signaling (Ko & Brandizzi, 2024; Waadt et al., 2022; Zhang et al., 2022). Among these, phytohormones play a pivotal role as intermediates that either amplify initial signals or initiate downstream signaling cascades to coordinate abiotic stress responses (Waadt et al., 2022). This led us to investigate whether genes responsive to phytohormones also participate in the transcriptional reprogramming triggered by abiotic stress.

We identified hormone‐responsive genes in sorghum through homology‐based BLAST searches, an approach widely used for comparative analyses (Hirsch et al., 2014; Kalanon & McFadden, 2008; van Holle et al., 2017). As queries, we used Arabidopsis (*Arabidopsis thaliana*) genes responsive to abscisic acid (ABA), 1‐aminocyclopropane‐1‐carboxylate (ACC, an ethylene precursor), brassinolide (BL), gibberellic acid (GA), and indole‐3‐acetic acid (IAA), based on published expression datasets (Goda et al., 2008). Applying stringent filtering (Figure S8a; “Materials and Methods” section), we identified 871 ABA‐, 88 ACC‐, 105 BL‐, 88 GA‐, and 245 IAA‐responsive homologs in the sorghum genome (Figure S8b; Data S10). The higher number of homologs compared with Arabidopsis likely reflects lineage‐specific genome duplications in sorghum (Paterson et al., 2009).

**Figure 3:**
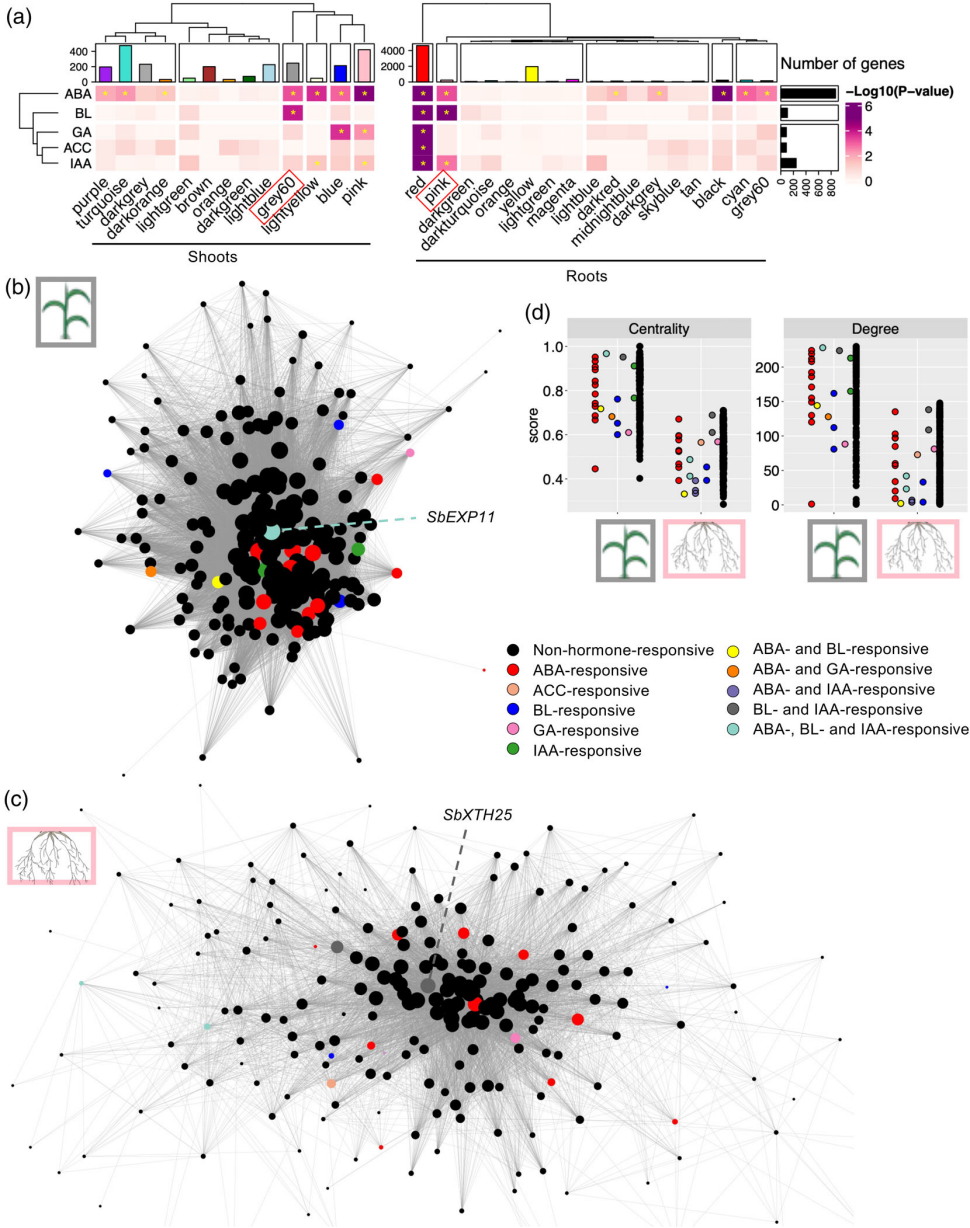
S‐gray60 and R‐pink are associated with phytohormone‐responsive genes yet display distinctive network traits. (a) Enrichment patterns of phytohormone‐responsive genes in co‐expression modules in shoots and roots at the right and left, respectively. The heatmap shows the various levels of statistical enrichment of the identified sorghum phytohormone marker genes. The vertical bar graph on the right side indicates the number of genes that belong to each set of hormone‐responsive genes. Statistically significant enrichment (*P* < 0.05 [−log_10_ *P* = 1.3]) is indicated by yellow asterisks. (b, c) Co‐expression networks of shoot‐gray60 (b) and R‐pink (c). Circle nodes are color‐coded according to responsiveness to hormones as indicated. All interactions with >0.2 thresholds of weight were included. The size of nodes is proportional to the degree within each network. Annotations of highly ranked hormone‐responsive genes are shown with green dashed lines. (d) Scatter plots showing two network traits, centrality and degree, of each gene within shoot‐gray60 and root‐pink. The color codes are the same as shown in (b, c).

These hormone‐responsive genes were significantly enriched across various co‐expression modules, with ABA‐responsive genes being the most widely enriched (Figure 3a). In contrast, ACC‐responsive genes were predominantly enriched in the red module in roots, consistent with the greater role of ABA in environmental stress responses compared with ethylene (Waadt et al., 2022). Notably, the S‐gray60 module showed significant enrichment for ABA‐ and BL‐responsive genes, while the R‐pink module was enriched for ABA‐, BL‐, and IAA‐responsive genes, suggesting that these modules may functionally link hormone response pathways via specific regulatory genes.

To explore the regulatory architecture of these modules, we constructed co‐expression network maps for S‐gray60 and R‐pink based on gene connectivity and interaction strength, overlaying hormone‐responsiveness annotations (Figure 3b,c). These network maps revealed densely connected gene clusters with many hormone‐responsive genes occupying central (hub) positions. For example, in S‐gray60, *Sobic.004G121900*, encoding an ABA/BL/IAA‐responsive encoding EXPANSIN 11 (*SbEXPA11*), had one of the highest connectivity scores (ranked 7th overall). Similarly, in R‐pink, *Sobic.007G086400*, a BL/IAA‐responsive gene encoding a xyloglucan endotransglucosylase/hydrolases 25 (SbXTH25), ranked 11th overall. These genes, designed as hormone‐responsive hub genes (HHGs), are likely to play key roles in orchestrating the tissue‐specific transcriptional suppression observed exclusively at the heat M1 phase. qRT‐PCR analysis confirmed their downregulation during this mid‐phase of heat stress (Figure S9). In Arabidopsis, AtEXPA11 and other EXPANSIN family members are known to mediate hormone and stress responses in non‐root tissues (Molina et al., 2021; Samalova et al., 2023) while *AtXTH* genes regulate lateral root development under stress conditions (Xu et al., 2020). Thus, the functional roles of these HHGs in tissue‐specific abiotic stress responses are likely conserved across monocots and dicots. Finally, we quantified network properties such as centrality (a measure of how close a node is to all others) and degree (the number of co‐expression links per gene). Consistent with their network structures (dense and compact for S‐gray60 versus broader and more diffuse for R‐pink), S‐gray60 exhibited higher centrality and degree values compared with those in R‐pink (Figure 3d). These findings suggest that despite similar expression patterns and shared enrichment of ABA‐ and BL‐responsive genes (Figures 2a and 3a), S‐gray60 and R‐pink establish distinct transcriptional architectures that contribute to the tissue‐specific stress responses.

### Identification of TFs upstream of HHGs

**Figure 4:**
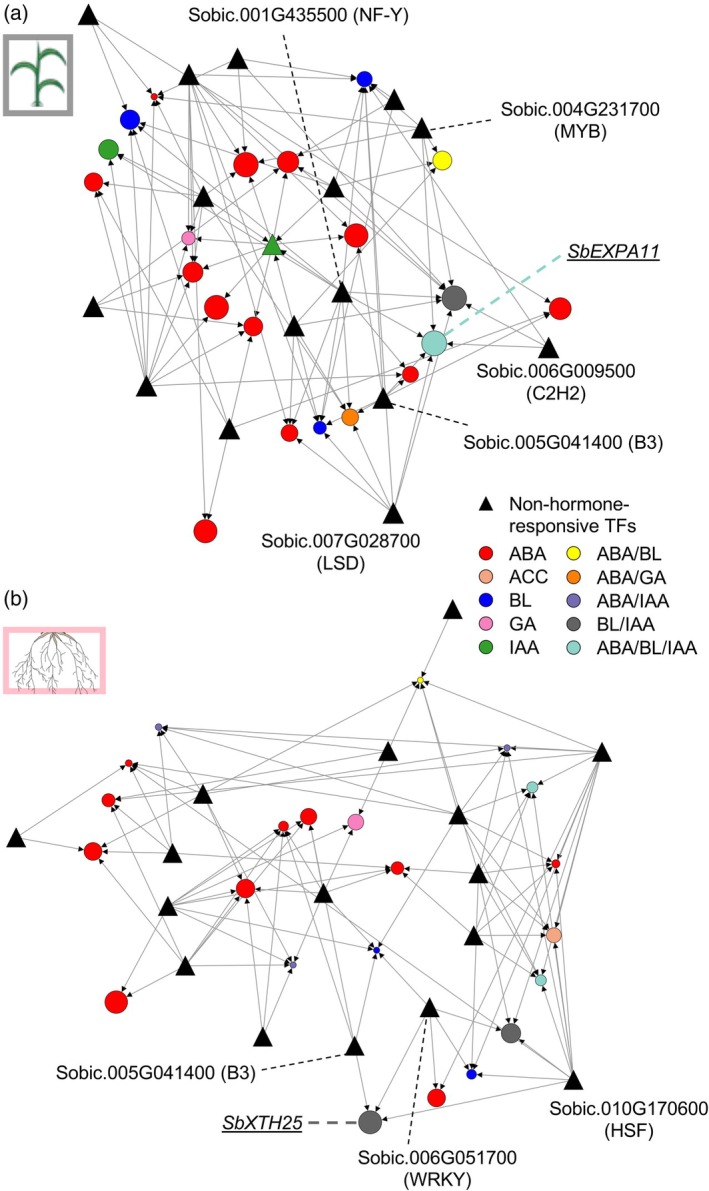
GRN mapping reveals TFs upstream of the hub gene in each S‐gray60 or R‐pink. GRNs showing predicted regulation of phytohormone‐responsive genes by TFs in S‐gray60 (a) and R‐pink (b), respectively. TFs that are predicted to regulate the top hub gene in the co‐expression networks (Figure 3b,c) are summarized within dashed boxes. Circle nodes are color‐coded according to responsiveness to hormones as indicated. The size of circle nodes in panels (c) and (d) is proportional to the number of co‐expression links shown in Figure 3(b) and (c), respectively. The color codes of nodes are the same as shown in Figure 3. Complete lists of TFs and input files are provided in Data S11.

We hypothesized that identifying TFs regulating the expression of HHGs could provide valuable insights into engineering tissue‐specific heat stress resilience in sorghum. To investigate the upstream regulators of HHGs, we applied a machine learning‐based approach using the GENIE3 algorithm (Huynh‐Thu et al., 2010), which infers GRNs from transcriptomic data (Ko & Brandizzi, 2025). We constructed separate GRNs for the S‐gray60 and R‐pink modules, each containing 16 TFs (Data S11), based on gene expression dynamics across multiple time points and stress conditions (Figure 4). Our GRN modeling revealed highly interconnected regulatory networks, capturing transcriptional cascades in which one TF regulates another TF. Notably, each HHG was predicted to be regulated by multiple upstream TFs. For example, *SbEXPA11* (in S‐gray60) was predicted to be targeted by C2H2, B3, LSD, NF‐Y, and MYB TFs, while *SbXTH25* (in R‐pink) was associated with WRKY, HSF, and B3 TFs.

**Figure 5:**
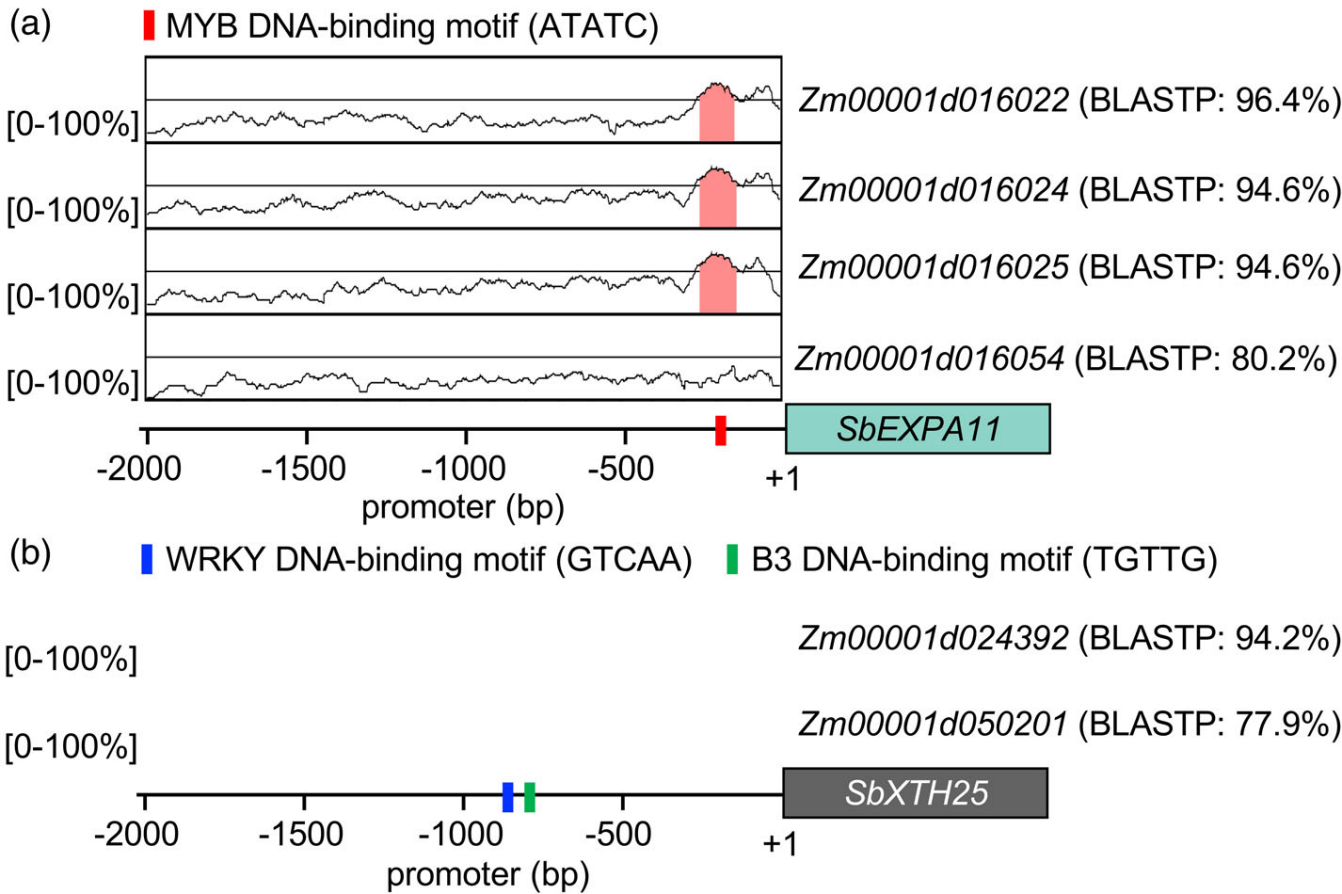
The promoters of *Sobic.004G121900* and *Sobic.007G086400* have conserved CREs for predicted upstream TFs. Conservation and divergence of the promoter sequences of *Sobic.004G121900* (a) and *Sobic.007G086400* (b) across sorghum and maize are illustrated through mVISTA plots. Regions shaded in salmon indicate >70% sequence similarity across 50‐bp windows. The blue, red, and green bars indicate MYB, WRKY, and B3 DNA‐binding motifs, respectively. Full sequence information is provided in Figure S10.

To validate these GRN predictions, we leveraged the evolutionary conservation of functionally important CREs. Given the close evolutionary relationship between sorghum and maize (*Zea mays* L.), which diverged approximately 12 million years ago (Swigonova et al., 2004; Wang et al., 2015), we used maize as a comparative model for cross‐species promoter analysis. Homologs of the HHGs were identified in maize: four for SbEXPA11 and two for SbXTH25 (Figure 5). We then examined the promoter sequence conservation using the VISTA algorithm (Frazer et al., 2004), comparing 2‐kb upstream regions of the maize homolog genes to the sorghum HHG promoters. For *SbEXPA11*, we identified a conserved 109‐nt promoter region shared among three closely related maize homologs (*Zm00001d016022*, *Zm00001d016024*, and *Zm00001d016025*) but not present in a more distantly related homolog (*Zm00001d016054*). This conserved region contains a MYB binding motif (ATATC), supporting our GRN prediction that the MYB TF (Sobic.004G231700) regulates targets *SbEXPA11* in the S‐gray60 module (Figure 5a; Figure S10a). Similarly, for *SbXTH25*, we identified two conserved promoter regions (413‐nt and 338‐nt) shared with a close maize homolog (*Zm00001d024392*), but absent in a more distant homolog (*Zm00001d050201*) (Figure 5b; Figure S10b,c). These regions included WRKY (GTCAA) and B3 (GCATG) binding motifs, consistent with our GRN predictions that WRKY and B3 TFs regulate *SbXTH25* (Figure 4b).

Together, these results indicate that shoot‐ and root‐specific GRNs are enriched for phytohormone‐responsive genes, coordinated by HHG (*SbEXPA11* and *SbXTH25*) and their predicted upstream regulators. These regulators appear to contribute to the transcriptional suppression observed at the M1 stage of heat stress and are likely involved in modulating key biological pathways. Overall, our integrative approach combining GRN modeling with comparative promoter analysis provides a robust framework for identifying high‐confidence TF candidates upstream of HHGs, key components of tissue‐specific regulatory networks underpinning stress resilience in sorghum.

## DISCUSSION

Climate change, including global warming and extreme weather, presents formidable challenges to plant survival and crop productivity. To adapt, plants reprogram their gene expression in stress‐, time‐, and tissue‐specific manners (Alvarez et al., 2021; Ko & Brandizzi, 2024; Swift & Coruzzi, 2017). Prior studies examined transcriptomes under drought, heat, and salinity in sorghum (Buchanan et al., 2005; Dugas et al., 2011; Fracasso et al., 2016; Johnson et al., 2007; Pardo et al., 2023; Varoquaux et al., 2019), but these investigations often focused on individual stressors, specific time points, or single tissue types. This approach has limited the ability to compare the functional impact of these key factors in parallel. Our study addresses this significant knowledge gap by generating a comprehensive transcriptomic resource that integrates stress, time, and tissue dynamics within a single experimental and computational framework.

Through co‐expression network modeling, we revealed the tissue specificity of abiotic stress‐inducible gene reprogramming. Specifically, we identified two co‐expression modules, one in shoots and one in roots, with nearly identical expression patterns but enriched in distinct phytohormone‐responsive genes. Our GRN modeling highlighted a hierarchical structure within each module, identifying specific candidate regulators of HHGs. Additionally, the promoter scanning algorithm allowed us to pinpoint upstream regulators of these HHGs, providing mechanistic insights into their regulation. While the GRN inference and promoter enrichment analyses presented here provide mechanistic insight into putative regulatory hierarchies, we emphasize that these predictions represent hypothesis‐generating outcomes for future experimental validation of individual TF–target relationships, for example using *in vivo*, *in vitro* DNA‐binding assays, or transient expression assays. Specifically, the goal of this study is to provide a high‐resolution transcriptomic and network‐level framework to guide downstream functional and physiological investigations. Our analyses prioritize the identification of stress‐responsive modules, hub genes, and regulatory hierarchies that represent testable hypotheses for future genetic and physiological studies aimed at dissecting causal links between transcriptional reprogramming and stress resilience.

Our findings demonstrate that tissue specificity is the dominant factor shaping abiotic stress‐responsive transcriptomic changes, with roots and shoots orchestrating distinct GRNs linked to similar biological pathways. This conclusion is supported by evidence from the model species Arabidopsis, maize, or rice (*Oryza sativa*), where tissue‐specific GRNs underlie numerous biological processes, including stress responses (Pérez‐Díaz et al., 2014), circadian regulation (James et al., 2008), seed development (Lu et al., 2013; Yi et al., 2019; Zhang et al., 2024), and vegetative growth (Movahedi et al., 2011). Additionally, spatially non‐overlapping regulatory modules mediate cell type‐level responses to environmental stress (Dinneny et al., 2008; Rich‐Griffin et al., 2020), underscoring the need for higher resolution studies. For instance, in Arabidopsis, roots comprise at least 14 cell types (Shahan et al., 2022), each likely governed by unique regulatory circuits critical for growth, development, or stress responses (Han et al., 2024; Jean‐Baptiste et al., 2019; Zhang et al., 2019). Advances in single‐cell genomics technologies could enable the dissection of these organ‐specific GRNs (Han et al., 2024; Marand et al., 2023). Identifying organ‐, tissue‐, and cell type‐specific target genes will facilitate the engineering of stress‐resilient crops while minimizing unintended growth penalties or pleiotropic effects associated with whole‐organism approaches.

Phytohormones play pivotal roles in plant responses to abiotic stress at molecular, cellular, and physiological levels (Waadt et al., 2022). ABA, for example, protects plants from osmotic and ionic stresses through ABA‐responsive element (ABRE)‐mediated transcriptional regulation. Arabidopsis mutants deficient in ABA are hypersensitive to dehydration (Nambara et al., 1998), while plants overexpressing ABRE‐binding TFs exhibit enhanced tolerance to multiple stresses (Kim et al., 2004). Similarly, auxin, a master regulator of growth, mediates morphological adaptations to elevated temperatures, salt stress, and drought through processes like stomatal closure (Jing et al., 2023). Our discovery that phytohormone‐responsive genes are highly enriched and ranked in tissue‐specific co‐expression modules aligns with these established roles. Notably, HHGs such as *SbEXPA11* in S‐gray60 and *SbXTH25* in R‐pink, together with their predicted upstream regulators, represent high‐priority candidates for targeted functional validation. The regulatory relationships proposed here provide a focused framework for future molecular and physiological studies aimed at linking transcriptional regulation to stress tolerance phenotypes.

As plants must execute precise gene expression changes in specific tissues and at the correct times to maintain cellular homeostasis under extreme environmental conditions, understanding the regulatory complexity and interplay among stress, time, and tissue factors is critical. Our study demonstrates that tissue specificity is the primary driver of gene reprogramming, operating through highly interconnected GRNs where HHGs and their regulators are predicted using advanced data modeling. By providing a foundational transcriptomic and network resource, this work establishes a framework for future genetic and physiological studies and supports the rational engineering of tissue‐specific regulatory hubs to enhance crop climate resilience while minimizing growth penalties. Such strategies hold significant promise for improving food security in a rapidly changing climate.

## MATERIALS AND METHODS

### Analysis of the relationship between sorghum yield and temperature changes in the USA

Sorghum yield data (element code: 5419) and temperature anomaly data from 1961 to 2022 for the United States (area code: 840) were obtained from the Food and Agriculture Organization of the United Nations (https://www.fao.org/home/en/). For temperature, we used July data from each year, calculated relative to a baseline climatology from 1951 to 1980. The Pearson correlation coefficient (PCC) was calculated using the cor.test() function in R (version 4.1.3). Results were visualized with the ggplot2 package (version 3.5.2). A complete list of data points is provided in Table S1.

### Plant growth and abiotic stress treatment

Seeds of the sorghum reference genotype, BTx623, were planted in a standard potting mix composed of the commercial soil products as follows: 40% General Propagation Mix, 20% Sure‐Mix, and 40% Redi‐Earth. Plants were grown in a Percival growth chamber under a 14‐h light/10‐h dark (14L:10D) photoperiod with light intensity of 600 μmol m^−2^ sec^−1^. The temperature was maintained at 30°C during the light period and 24°C during the dark period. Plants were arranged in a randomized design and rotated daily to minimize positional effects. Drought stress was initiated by withholding water starting at 8 DAP at 9:00 am. We hand‐watered plants by providing water in trays so that the soil absorbed water naturally. We drained the excessive water 1 h later. Heat stress treatment began at 13 DAP, when plants were exposed to 45°C at 9:00 am with regular daily watering. For salinity stress, plants were watered with 200 mM NaCl (Sigma‐Aldrich, St. Louis, MO) daily from 13 DAP at 9:00 am. Control plants were maintained under standard growth conditions without stress treatments. Above‐ground (shoot) and below‐ground (root) tissues were harvested at 13 DAP (3:00 pm, 6 h after stress onset), 14 DAP (9:00 am, 24 h), 15 DAP (9:00 am, 48 h), and 16 DAP (9:00 am, 72 h) for each set (i.e., control, drought, heat, or salinity). Tissues were immediately frozen in liquid nitrogen. For root samples, soil particles were removed by briefly rinsing with running water, followed by gentle blotting with paper towels to remove excess moisture before freezing. Each biological replicate consisted of pooled tissues from two individual seedlings, with three biological replicates per sample.

### Soil water content measurement

Drought stress experiments, identical to those described above and in Figure S2(a), were conducted to quantify soil water content using five biological replicates. Soil pots from which all above‐ground sorghum tissues had been removed were collected at 8, 13, and 16 DAP under drought conditions. For each replicate, soils from two pots were pooled and weighed using a balance to determine the “wet weight” (WW). Immediately after weighing, the pots were incubated in a drying oven at 75°C for 48 h to remove all moisture. The dried pots were then weighed to obtain the “dry weight” (WD). Soil water content for each replicate was calculated using the formula: Soil water content (%) = [(WW − WD)/WD] × 100.

### RNA extraction and RNA sequencing (RNA‐seq) analyses

The frozen tissues were ground to a fine powder in liquid nitrogen using a mortar and pestle. Total RNA was extracted from the ground tissue powder using the NucleoSpin RNA Plant kit (MACHEREY‐NAGEL, Düren, Germany) according to the manufacturer's instructions. RNA‐seq libraries were constructed using Illumina's TruSeq Stranded mRNA HT sample prep kit (Illumina, San Diego, CA, USA) and sequenced in paired‐end mode on the Illumina NovaSeq S4 platform (150‐nt) at the Joint Genome Institute. The quality of raw reads was evaluated using FastQC (version 0.11.5). Reads were cleaned for quality and adapters with Cutadapt (version 1.8.1) (Martin, 2011) using a minimum base quality of 20 retaining reads with a minimum length of 30 nucleotides after trimming. Quality‐filtered reads were aligned to the sorghum reference genome (v3.1.1) using TopHat (version 2.0.14) (Kim et al., 2013) with Bowtie (version 2.2.4) (Langmead & Salzberg, 2012) used solely for reference genome indexing. Alignments were performed using a 10‐bp minimum intron length and 15 000‐bp maximum intron length. Fragments per kilobase exon model per million mapped reads (FPKM) were calculated using the sorghum genome model annotation (v3.1.1) with Cufflinks (version 1.3.0) (Trapnell et al., 2010). Per‐gene read counts were measured using HTSeq (version 0.6.1p1) (Anders et al., 2015) in the union mode with a minimum mapping quality of 20 with stranded = reverse counting. Differential gene expression analysis was performed in each sample relative to the mock control using DESeq2 (version 1.36.1) (Love et al., 2014) within R (version 4.1.3). Genes of which the total count across conditions and replicates (i.e., control‐1, control‐2, control‐3, stress‐1, stress‐2, stress‐3) in each genotype is <100 were not included in the analysis. All genes analyzed were visualized for each genotype in volcano plots using R package EnhancedVolcano. Differentially expressed genes (DEGs) were obtained based on adjusted *P*‐value <0.05 and absolute Log_2_FC >1. GO enrichment analysis was performed using agriGO (version 2.0) (http://systemsbiology.cau.edu.cn/agriGOv2/) (Tian et al., 2017) with a false discovery rate‐adjusted *P* < 0.05 (hypergeometric test with Bonferroni correction). Biological process GO categories were using a background gene set comprising 15 420 annotated sorghum genes associated with 3153 GO terms, derived from Phytozome v11 (locus ID version 3.1), corresponding to the same genome annotation used for RNA‐seq read mapping and downstream analyses. Enriched GO terms were visualized as heatmaps using the R package ComplexHeatmap (version 2.14.0) (Gu et al., 2016). All intersections of DEGs shown in this study were identified and visualized using R package ggVennDiagram (version 1.5.2). For the intersection analysis of DEGs found in this study with the ones from Johnson et al. (2014), we obtained the full list of DEGs from the original paper and then coverted the v1 gene IDs to v3 ones to be comparable with our DEGs using the SorGSD gene conversion tool (https://ngdc.cncb.ac.cn/sorgsd/geneconversion).

### Principal component analysis (PCA)

Raw gene‐level read counts generated by HTSeq were used as input. To reduce noise from lowly expressed genes, we retained genes with at least 10 reads in a minimum of three samples across the dataset. Variance stabilization was applied using the DESeq2 variance‐stabilizing transformation (vst(), blind = TRUE), which accounts for mean–variance dependence in count data and enables cross‐sample comparability. PCA was computed on the variance‐stabilized expression matrix using the plotPCA() function in DESeq2. PCA was performed at three levels: (i) using all samples jointly to evaluate the relative contributions of tissue, stress, and temporal effects to global transcriptomic variation; and (ii) separately for root and shoot samples to examine stress‐ and time‐dependent responses within each tissue. For tissue‐specific PCA, only samples from the corresponding tissue were included, and variance stabilization and PCA were performed independently. PCA results were visualized by mapping experimental factors (tissue, stress, and time) onto the same PCA coordinates to facilitate interpretation of the dominant drivers of variation.

### qRT‐PCR analysis

cDNA was synthesized from 1 μg of DNase I‐treated total RNA using the iScript cDNA Synthesis Kit (Bio‐Rad, Hercules, CA, USA), following the manufacturer's instructions. For qRT‐PCR, 2 μl of the 10‐fold diluted cDNA was used as the template in reactions containing gene‐specific primers and Fast SYBR™ Green Master Mix (Applied Biosystems, Foster City, CA, USA). Amplification was performed on ABI 7500 Real‐Time PCR System (Applied Biosystems). Relative expression was normalized to *Acyl Carrier Protein 2* (*ACP2*) (Sudhakar Reddy et al., 2016). The PCC and significance level between qRT‐PCR and RNA‐seq data were calculated using the cor.test() function in R (version 4.1.3). Results were visualized with the ggplot2 package (version 3.5.2). A complete list of primers is provided in Table S2.

### Co‐expression network analyses

For co‐expression network analysis, we constructed tissue‐specific union sets of DEGs, defined as genes that were significantly differentially expressed at least at one time point under any stress condition. These union DEGs sets comprised 2447 genes in shoots and 8678 genes in roots and were used as input for network construction. Co‐expression networks were generated using the WGCNA R package (Langfelder & Horvath, 2008). As the primary objective of this study was to identify transcriptional reprogramming in response to abiotic stress, we used log_2_‐transformed DESeq2‐normalized fold changes (stress versus control) as input, rather than raw or normalized expression values. Fold change‐based inputs directly capture stress‐induced regulatory responses and minimize contributions from constitutive expression differences. Restricting the analysis to DEGs further reduces background noise from non‐responsive or lowly expressed genes, thereby enhancing the robustness and biological interpretability of co‐expression modules, an approach that has been widely adopted in RNA‐seq‐based WGCNA studies (Ko & Brandizzi, 2023, 2025; Lanver et al., 2018; Mutinda et al., 2023; Xing et al., 2022; Zhang et al., 2023). For selecting soft‐power threshold, we first run the scale‐topology fit index analysis to identify the value that passes 0.9 Scale‐free topology model fit, which was 32 in both tissues (Figure S6). Since less than 30 for signed networks is generally considered a reasonable power, instead of selecting 32, we followed the instruction that soft‐thresholding power 18 is recommended for signed networks with less than 20 samples. Thus, a soft‐power threshold of 18 was selected to create a signed network of a Spearman correlated matrix. Topographical overlap matrices (TOMs) were constructed using TOMsimilarityFromExpr() function with a default parameter. The TOM scores were used as edge weights in the analysis. Co‐expression modules were constructed through hierarchical clustering of the TOM distance using flashClust() function with (method=“average”). Modules were derived using the cutreeDynamic() function with 30 of minimum module size, and similar modules were merged into a single module using mergeCloseModules() function with (cutHeight = 0.25). Network visualization was achieved using the Cytoscape software (version 3.6.1) with a cutoff of the weight parameter obtained from the WGCNA, set at 0.2. We previously provided the detailed step‐by‐step instructions for WGCNA in a GitHub depository (https://github.com/DaeKwan‐Ko/WGCNA) (Ko & Brandizzi, 2023).

### Identification of sorghum homologs of phytohormone marker genes

To identify sorghum homologs of hormone‐responsive genes, we first retrieved genes responsive to abiotic stress‐related phytohormones (ABA, ACC, BL, GA, and IAA) from Arabidopsis based on table S5 (high‐stringency data) of Goda et al. (2008). We then obtained the corresponding protein sequences from Phytozome (version 13), resulting in 483 proteins for ABA, 43 for ACC, 41 for BL, 51 for GA, and 149 for IAA. For genes with multiple isoforms, we selected the longest isoform as the representative using a custom Perl script. Next, we performed BLASTP searches for each hormone‐specific Arabidopsis protein set against the sorghum proteome (version 3.1.1), using an E‐value cutoff of 1.0 × 10^−20^. BLASTP hits with less than 50% identity or 50% sequence coverage were excluded. This filtering yielded 871 ABA‐, 88 ACC‐, 105 BL‐, 88 GA‐, and 244 IAA‐inducible sorghum genes. We assessed the statistical enrichment of these hormone‐responsive sorghum genes across co‐expression modules using the GeneOverlap R package (version 1.40.0).

### Cistrome analysis

To assess DNA‐binding motif enrichment in the promoters of co‐expression module genes (S‐gray60, R‐pink, S‐pink, and R‐tan), we retrieved position weight matrices for 566 Arabidopsis TFs from the Plant Cistrome Database (http://neomorph.salk.edu/dev/pages/shhuang/dap_web/pages/index.php) to use as *a priori* motifs of interest. We then performed motif enrichment analysis using Analysis of Motif Enrichment (AME) (McLeay & Bailey, 2010) on 1‐kb upstream promoter regions of the sorghum module genes, obtained via BioMart from the sorghum genome annotation (version 3.1.1; https://phytozome‐next.jgi.doe.gov/biomart). As controls, we extracted 1‐kb promoter sequences from an equal number of randomly selected sorghum genes for each module. Enrichment scores were visualized as a heatmap using the R package ComplexHeatmap (version 2.14.0) (Gu et al., 2016).

### GRN construction

For GRN construction, Log_2_‐transformed DESeq2‐normalized fold changes (stress/control) served as input. We downloaded the full list of TFs in sorghum from the Plant TF Database (http://planttfdb.gao‐lab.org/index.php). Among the full list, we found 16 TFs in each of S‐gray60 and R‐pink modules. With the expression profiles and the lists of TFs, we constructed GRNs using GENIE3 (Huynh‐Thu et al., 2010) and identified top 1000 TF interactions with module genes from which we further narrowed down to those of hormone‐responsive genes. The resulting TF–target gene interactions data were integrated with the co‐expression interaction data generated by WGCNA to create the GRNs. We visualized GRNs constructed in this study using the Cytoscape software (version 3.6.1) (Shannon et al., 2003).

### Promoter analyses

We searched homologs of SbEXPA11 and SbXTH25 using BLASTP from *Z. mays* (RefGen_V4) using BLASP in Phytozome (v13). This identified the three homologs of SbEXPA11 and two homologs of SbXTH25. We kept the ones that had the lowest E‐value and the longest among the isoforms. Then, we obtained the 2‐kb promoter sequence of each homolog gene from the sorghum genome model annotation (v3.1.1) using Biomart (https://phytozome‐next.jgi.doe.gov/biomart). We aligned the 2‐kb promoter sequences of the maize homolog genes to that of either *SbEXPA11* or *SbXTH25* using mVISTA LAGAN alignment (https://genome.lbl.gov/vista/mvista/submit.shtml) (Frazer et al., 2004) with the following parameters (Min_Y Minimum Y value on the VISTA plot, 20; Min_Id Minimum conservation identity, 70; Min_Length Minimum length for a CNS, 100).

### Statistical analysis

All statistical analyses were performed using R (versions 4.1.2 to 4.4.2). For Figure S1(b), differences among multiple groups were assessed using one‐way analysis of variance (ANOVA), followed by Duncan's multiple range test to determine significance (*P* < 0.05). Exact sample sizes (*n*) are indicated in the corresponding figure panel. Gene overlap analysis (Figures S4 and S5a–d) and hormone‐responsive gene enrichment (Figure 3a) were evaluated using hypergeometric test and Fisher's exact test, respectively.

## AUTHOR CONTRIBUTIONS

DKK and FB conceived the project and designed experiments and research plan; DKK performed experiments and data analysis; FB supervised the project; DKK and FB interpreted the data; DKK wrote the manuscript draft; DKK and FB edited the manuscript.

## CONFLICT OF INTEREST

The authors declare no conflicts of interest.

## Supporting information


**Figure S1.** Impact of temperature increase on sorghum production in the US.
**Figure S2.** The RNA‐seq analysis is designed to reveal tissue, temporal, and stress specificity.
**Figure S3.** Gene expression changes in each tissue at each timepoint under different abiotic stress conditions.
**Figure S4.** Intersection of DEGs identified among stress types.
**Figure S5.** Strong concordance with previously identified stress‐responsive genes and high reproducibility of our dataset.
**Figure S6.** Processing data of WGCNA.
**Figure S7.** Co‐expression network analysis identifies modules of abiotic stress‐inducible DEGs in sorghum shoots and roots.
**Figure S8.** Identification of phytohormone marker genes in sorghum.
**Figure S9.** qRT‐PCR validation of SbEXPA11 and SbXTH25 expression during the heat stress time course.
**Figure S10.** TF‐binding motifs in the conserved promoter regions of *SbEXPA11* and *SbXTH25*.

**Table S1.** Data points visualized in Figure S1.
**Table S2.** List of primers used in this study.

**Data S1.** Normalized read values (FPKM) for all genes annotated in the sorghum genome across all samples.
**Data S2.** A complete list of DEGs obtained in this study at the timepoints in each tissue under each stress.
**Data S3.** GO term analyses of the 22 and 183 DEGs responsive to all stressors in shoots and roots, respectively.
**Data S4.** Log2FC values for DEGs in shoots under stress conditions compared to the control.
**Data S5.** Log2FC values for DEGs in roots under stress conditions compared to the control.
**Data S6.** List of genes grouped into coexpression modules identified by WGCNA in shoots.
**Data S7.** List of genes grouped into coexpression modules identified by WGCNA in roots.
**Data S8.** GO term enrichment analysis for each coexpression module identified in shoots.
**Data S9.** GO term enrichment analysis for each coexpression module identified in roots.
**Data S10.** Hormonsive genes in Arabidopsis and their sorghum orthologs. Genes are listed in alphabetical order.
**Data S11.** List of TFs in the S‐gray60 and R‐pink coexpression modules, along with the input data used to construct the GRNs presented in Figure 4(a) (shoots) and Figure 4(b) (roots).

## Data Availability

All data supporting the findings of this study are available within this paper and its Supplementary Materials files. The raw data of RNA‐seq have been deposited to the National Center for Biotechnological Information Sequence Read Archive and are accessible via BioProject accession codes PRJNA1188145 (https://www.ncbi.nlm.nih.gov/bioproject/PRJNA1188145). The processed data of RNA‐seq analyses are available in Supplementary Data. Code availability: The scripts used in this study are available in GitHub (https://github.com/DaeKwan‐Ko/abiotic_bulkrnaseq).
