# Supplementary material for: A tissue‐resolved, network‐based transcriptomic framework for abiotic stress responses in sorghum
Source: Plant J. 2026 Mar 29;126(1):e70834. doi: 10.1111/tpj.70834 (PMC13033392; doi:10.1111/tpj.70834)
Supplement: Supplementary file 1 — Figure S1. Impact of temperature increase on sorghum production in the US. Figure S2. The RNA‐seq analysis is designed to reveal tissue, temporal, and stress specificity. Figure S3. Gene expression changes in each tissue at each timepoint under different abiotic stress conditions. Figure S4. Intersection of DEGs identified among stress types. Figure S5. Strong concordance with previously identified stress‐responsive genes and high reproducibility of our dataset. Figure S6. Processing data of WGCNA. Figure S7. Co‐expression network analysis identifies modules of abiotic stress‐inducible DEGs in sorghum shoots and roots. Figure S8. Identification of phytohormone marker genes in sorghum. Figure S9. qRT‐PCR validation of SbEXPA11 and SbXTH25 expression during the heat stress time course. Figure S10. TF‐binding motifs in the conserved promoter regions of SbEXPA11 and SbXTH25. [file TPJ-126-0-s002.pdf]

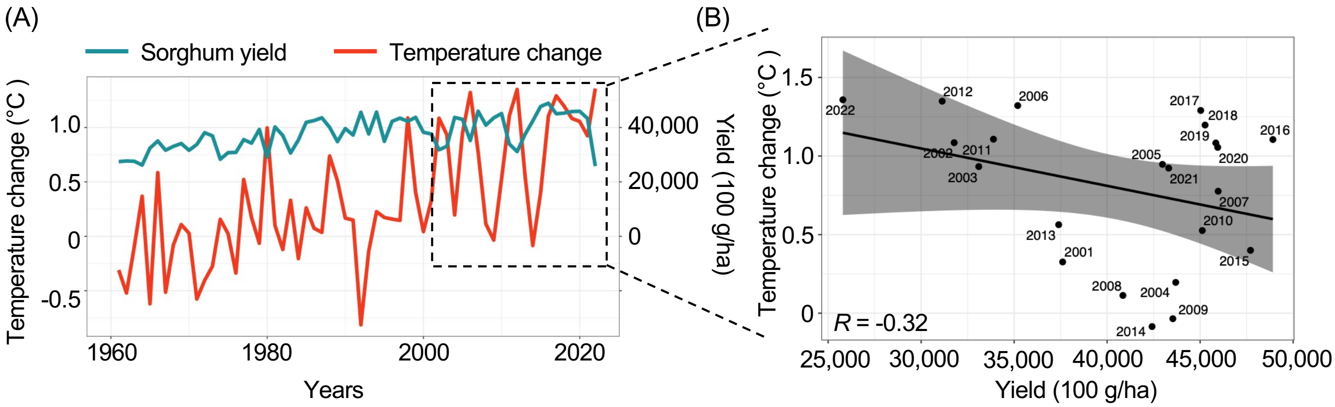

Figure S1. Impact of temperature increase on sorghum production in the US. (A) Historical data depicting sorghum production (teal) and temperature changes (red) in the US during July, compared to a baseline climatology from 1951 to 1980, for the years 1961 to 2022. (B) Negative correlation between sorghum yield and temperature increase from 2001 to 2022. The Pearson Correlation Coefficient is indicated in the bottom left corner. Sorghum yield and temperature change data was obtained from https://www.fao.org/home/en/.

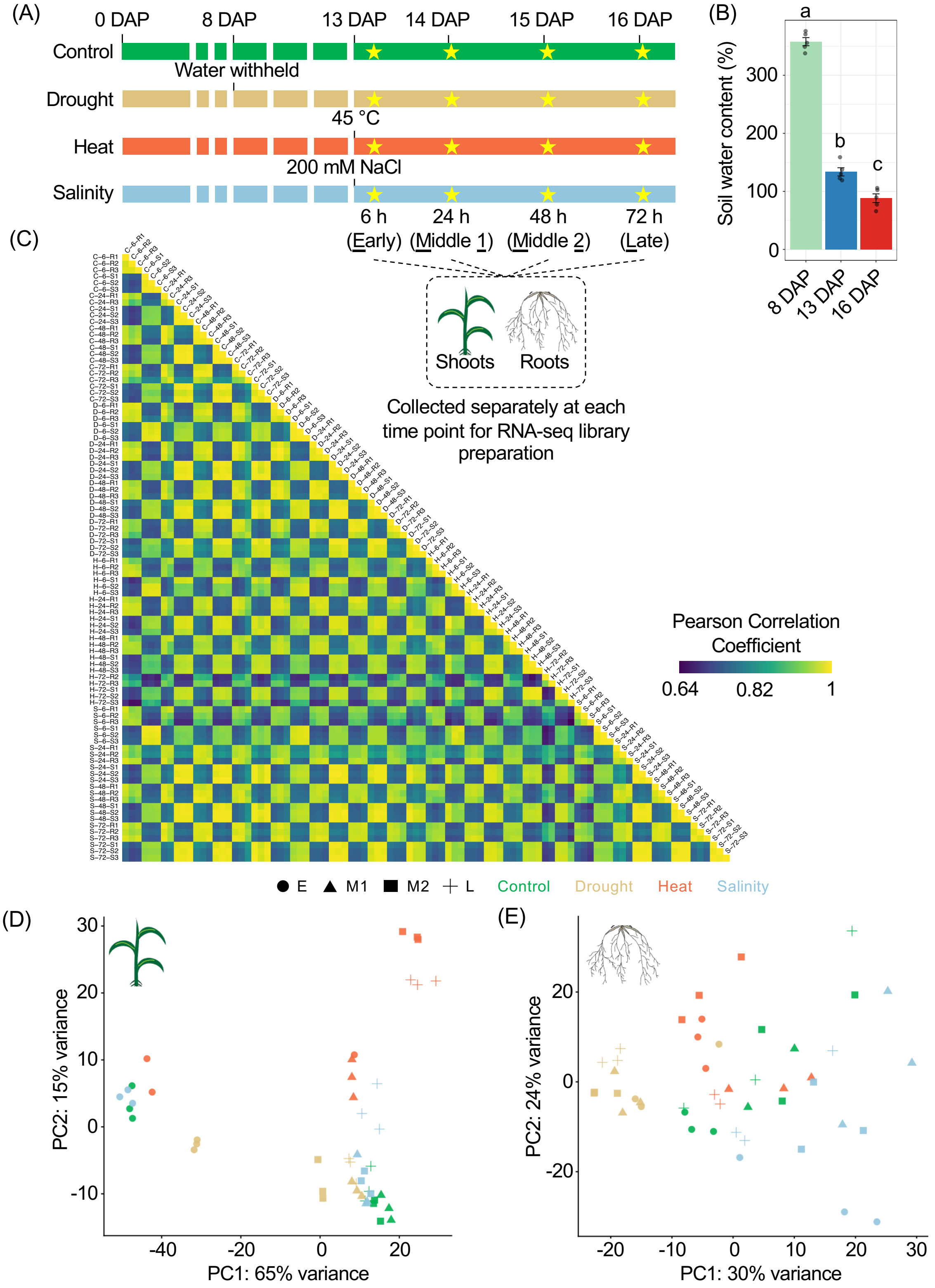

Figure S2. The RNA-seq analysis is designed to reveal tissue, temporal, and stress specificity. (A) Schematic view of the experimental design. Yellow stars indicate sample harvesting (E, early; M1, early middle; M2, late middle; L, late). DAP, days after planting. (B) Soil water contents under drought stress. The relative water contents in soil pots were measured at 8 DAP, 13 DAP, and 16 DAP. Means ± s.e.m.; n = 5 biological replicates. One-way ANOVA followed by Duncan's multiple range test ($P < 0.05$) was used to analyze the significance of differences among the multiple samples. Means with the same letter are not significantly different. The experiments were independently repeated twice with similar results. (C) High reproducibility of RNA-seq dataset in this study. Heatmap shows the Pearson correlation coefficient (PCC) of gene expression for all samples analyzed in this study. Genes with fragments per kb exon model per million mapped fragments (FPKM) > 1 in all samples were $\log_2$, +1 transformed before the analyses. The color scale indicates the Pearson correlation coefficient. The first letter of each sample indicates the corresponding treatment as follows: C, control; D, drought; H, heat. S, salinity. The second letter of each sample indicates the corresponding time point. The last number of each sample indicates the order of biological replicate. (D,E) PCA of shoot (D) and root (E) transcriptomes under abiotic stress. PCA of variance-stabilized gene expression values was performed using shoot samples only. Each point represents an individual biological replicate. Samples are colored by stress condition and shaped by time point. The percentages of variance explained by PC1 and PC2 are shown on the axes.

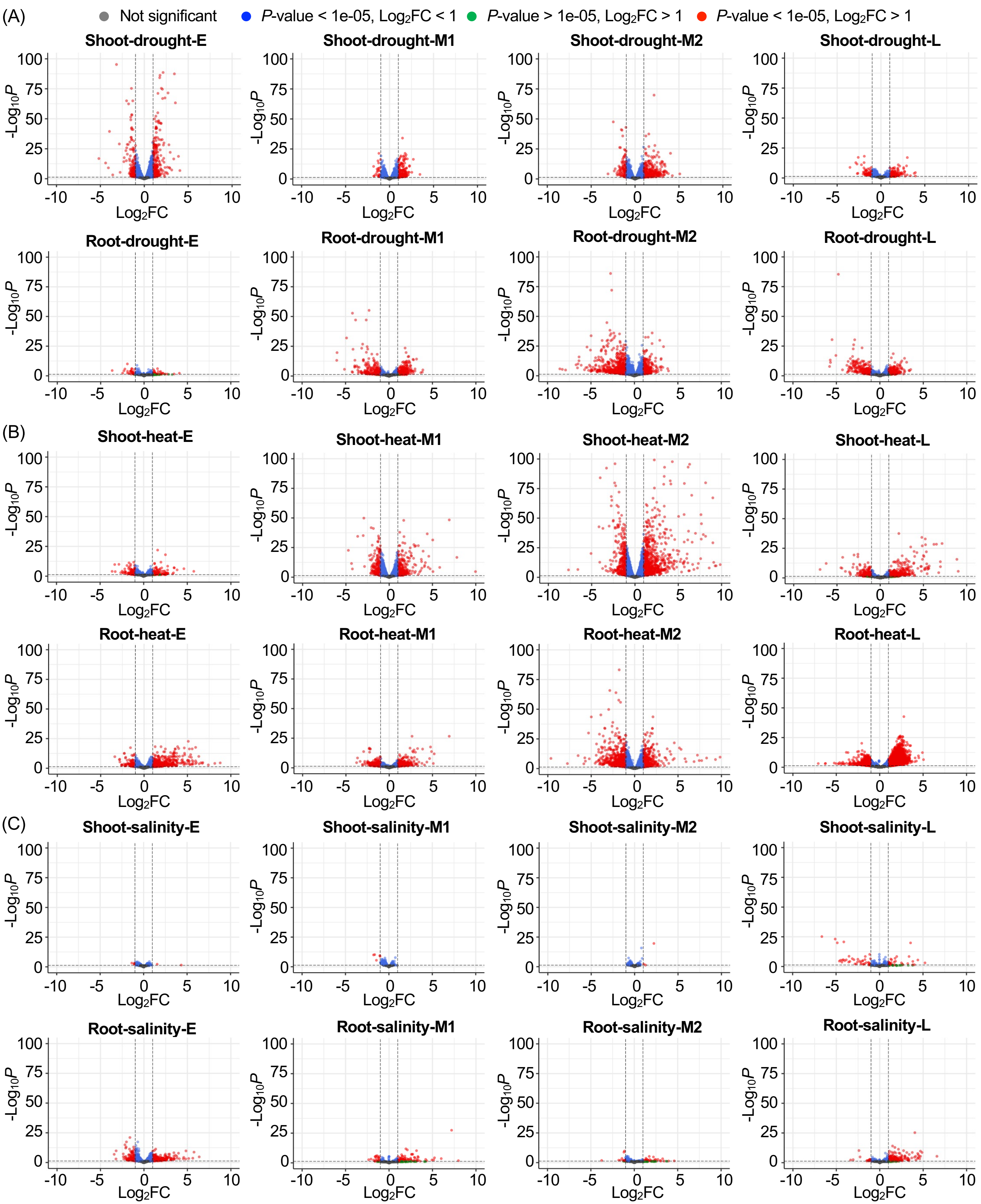

Figure S3. Gene expression changes in each tissue at each timepoint under different abiotic stress conditions. Volcano plots illustrate global normalized gene expression changes, as determined by DESeq2, for drought (A), heat (B), and salinity (C) stress. Each plot corresponds to a specific tissue and timepoint, with sample names indicated at the top of the respective plots.

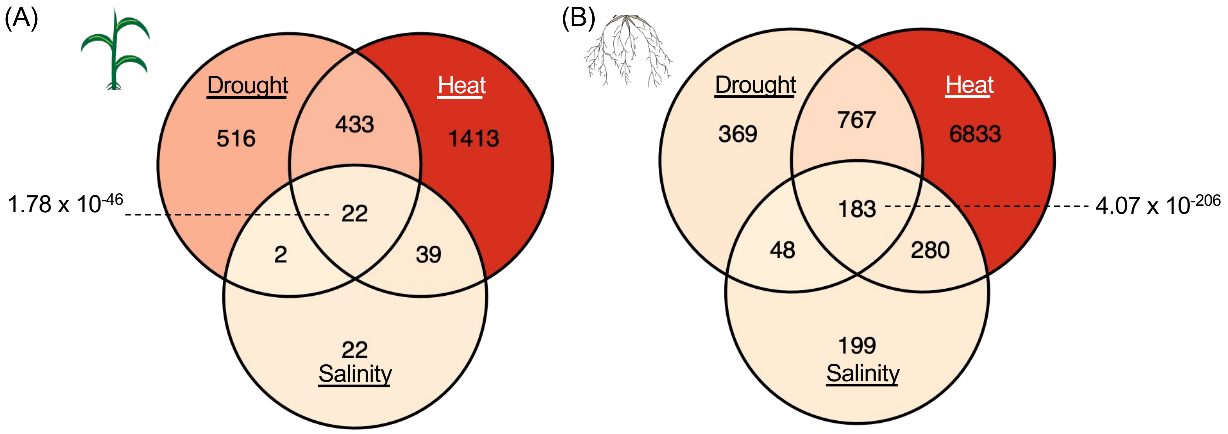

Figure S4. Intersection of DEGs identified among stress types. Venn diagrams showing the intersection of DEGs across drought, heat, and salinity stress conditions in shoots (A) and roots (B). The full list of DEGs is provided in Data S2. In all panels, the intensity of the red background in each region of the diagram indicates the number of DEGs, with deeper red representing higher counts. Statistical significance of the overlaps was assessed using the hypergeometric test and shown in each intersected region.

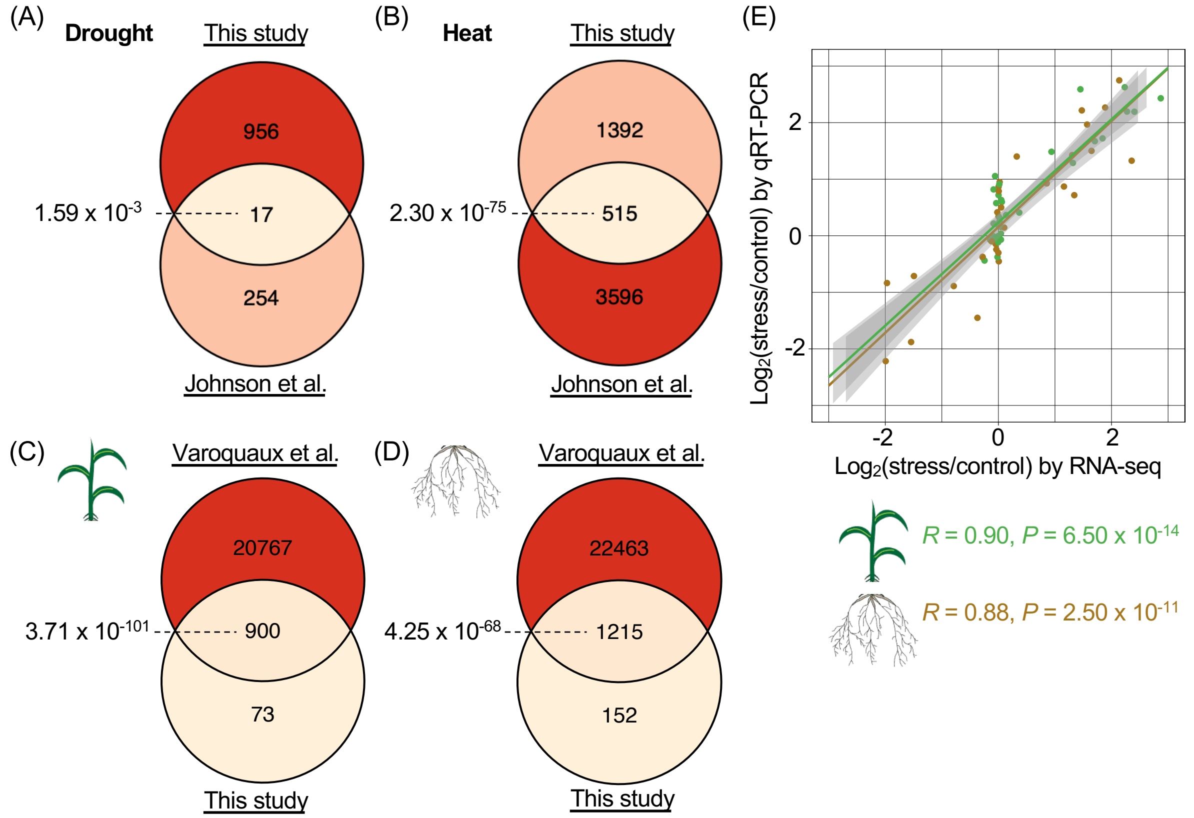

Figure S5. Strong concordance with previously identified stress-responsive genes, and high reproducibility of our dataset. (A,B) Venn diagrams showing the overlap of DEGs between this study and Johnson et al. under drought (A) and heat (B) stress. Since Johnson et al. analyzed shoot tissues, only shoot-derived DEGs from our dataset were included. (C,D) Venn diagrams illustrating the intersection of drought-responsive DEGs between this study and Varoquaux et al. in shoot (C) and root (D) tissues. In all diagrams, the intensity of the red shading corresponds to the number of DEGs within each region—darker red indicates a higher count. Statistical significance of the overlaps was calculated using the hypergeometric test and is indicated in each intersecting region. (E) qRT-PCR validation of differential expression for known stress marker genes (three genes per stress condition). A total of 69 Log_2FC values were obtained from qRT-PCR using the same RNA samples analyzed by RNA-seq. The scatter plot shows Pearson correlation coefficients between RNA-seq and qRT-PCR values for each tissue. Marker gene expression was assessed across multiple time points and tissues. Green and brown dots indicates shoots and root samples, respectively. Relative expression was normalized to *ACP2*. A full list of genes and primer sequences is available in Table S2.

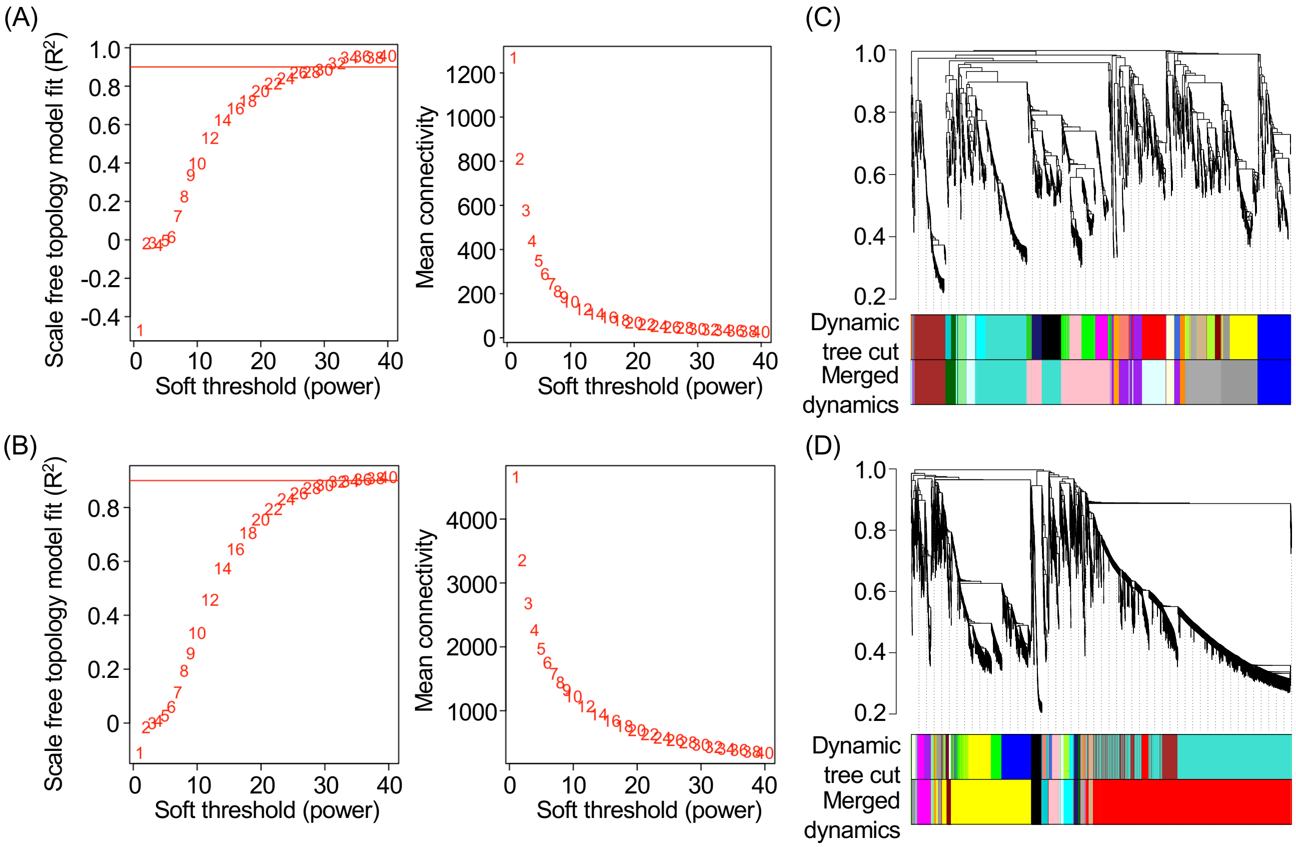

Figure S6. Processing data of WGCNA. (A,B) Network topology for soft threshold power. Plots of both scale-free fit index as a function of soft threshold power and mean connectivity as a function of soft threshold power are displayed for shoot (A) and root (B). Red lines in scale-free fit index plots indicate 0.9. (C,D) Hierarchical cluster dendrograms showing coexpression modules identified in shoot (C) and root (D).

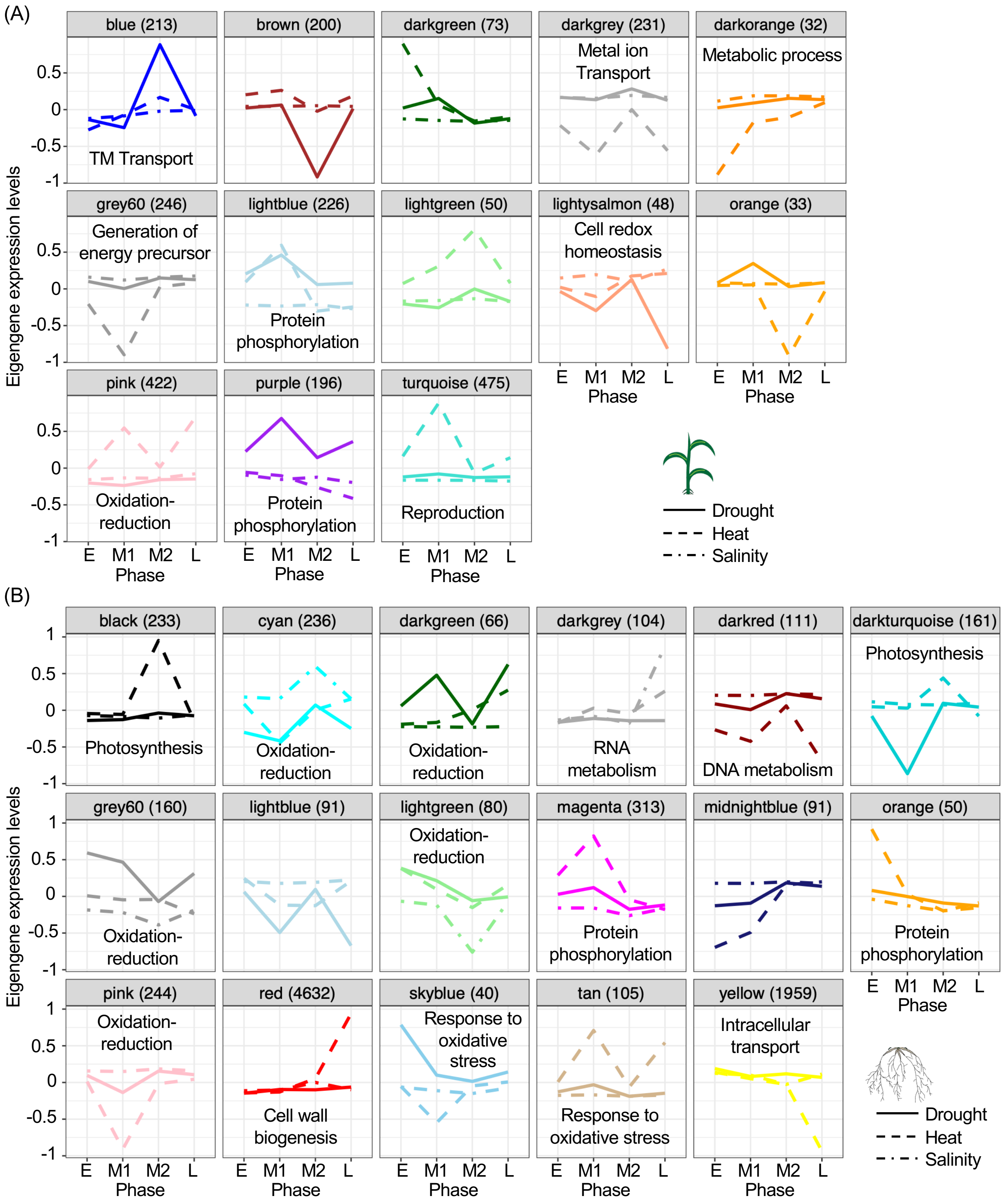

Figure S7. Coexpression network analysis identifies modules of abiotic stress-inducible DEGs in sorghum shoots and roots. DEGs from shoots (A) and roots (B) were analyzed using WGCNA to identify coexpression modules based on expression similarities across drought, heat, and salinity conditions. Each graph represents the module eigengene expression pattern across the stress phases, with the y-axis denoting the stress phase and the x-axis displaying the conditions. Modules are named according to their WGCNA-assigned color codes, shown at the top of each panel, with the number of genes in each module indicated in parentheses. The most representative GO term for each module is displayed, except for modules with no significantly enriched GO terms.

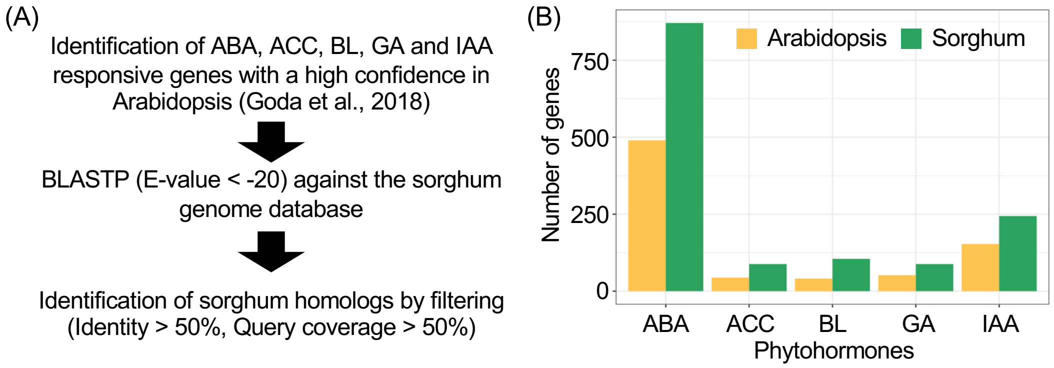

Figure S8. Identification of phytohormone marker genes in sorghum. (A) Schematic representation of the workflow used to identify sorghum homologs of phytohormone marker genes. ABA, abscisic acid. ACC, 1-aminocyclopropane-1-carboxylate. BL, brassinolide. GA, gibberellin acid. IAA, Indole-3-acetic acid. (B) Bar graph showing the number of phytohormone-responsive marker genes in Arabidopsis and their homologs identified in sorghum for each phytohormone.

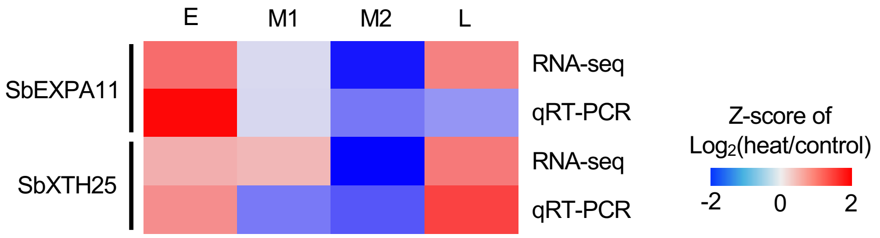

Figure S9. qRT-PCR validation of *SbEXPA11* and *SbXTH25* expression during the heat stress time course. qRT-PCR was conducted using the same RNA samples used for RNA-seq analysis. Relative expression levels were normalized to *ACP2*. Log_2-transformed fold-change values (heat vs. control) were Z-score scaled to allow direct comparison between RNA-seq and qRT-PCR results. Primer sequences used for qRT-PCR are listed in Table S2.

(A) GCGTCTCTCTTGTGGATTGCACCTTAAGCCATGCATATGCTGGGGCTGCACATAGCCATAGATATGTGCACGCTAACT
AGTGGATTACCAGGCTTGCTTCGTTCGACGC

(B) AAAGTTTGCAGTGCGCAAATATAAACAGTAATTGGTTGATTAAAAAAGTTCTGTTATGGTCGTAAAGTGTGCATGGACAT
GTGATCGTCAGGTGACTTGCCTGTTTGACTCCGCATCATTTGATTTCTTTTCTGAGCAACACTTGCATTAGTTTCTAAAC
CTGAGGAATTAATTCTTTTTAATAACCTCTTAGATCTGCCAGCAACTTACTCGCGTATAGCAAACCTGGTTTCTGTACAA
TATCATTGAATTGAAATAAATTAAGAGGGCAAGTGAAAATTTTCAATAACTTCAGAACACTATTATTATGATTTACAAGGC
AGGGAACAGGGACAATGCCACAAGTTCTATGCCATAACTTCAGAACCGTACATGTGGCTTTCTTTCAAGAGCATAATAA
TAAGCAGAGCTCA

(C) AAGCATGGCCTGGGCCTGATTAGGTTGGCAGGAGGCACCGTGGGGCGGCTAGCTATCCTGCTCAAGCTTTAATTCCA
GCACTTGAAGTTGAATCTTTATACTCGCTTATTCAGAAGCCAGCCATTCTGCCCAGTTTATTATGCCATCCAAGAAACAA
AGTCCATACCTAGTTAGATTCTATTCAGACTTGGACACTCTGCTC

MYB DNA-binding motif (ATATC) WRKY DNA-binding motif (GTCAA) B3 DNA-binding motif (GCATG)

Figure S10. TF-binding motifs in the conserved promoter regions of *SbEXPA11* and *SbXTH25*. DNA sequences of the conserved promoter regions of *SbEXPA11* (A) and *SbXTH11* (B,C) are shown, with TF-binding motifs highlighted. The corresponding regions are illustrated in Fig. 5.
