# Supplementary material for: A tissue‐resolved, network‐based transcriptomic framework for abiotic stress responses in sorghum
Source: Plant J. 2026 Mar 29;126(1):e70834. doi: 10.1111/tpj.70834 (PMC13033392; doi:10.1111/tpj.70834)
Supplement: Supplementary file 2 — Table S1. Data points visualized in Figure S1. Table S2. List of primers used in this study. [file TPJ-126-0-s003.docx]

**Table S1.** Data points visualized in Figure S1.

| Year | Sorghum yield (hg/ha) | Temperature change (°C) |
| --- | --- | --- |
| 1961 | 27442 | -0.31 |
| 1962 | 27679 | -0.521 |
| 1963 | 27573 | -0.092 |
| 1964 | 26181 | 0.368 |
| 1965 | 32405 | -0.62 |
| 1966 | 35026 | 0.585 |
| 1967 | 31633 | -0.514 |
| 1968 | 33046 | -0.079 |
| 1969 | 34097 | 0.108 |
| 1970 | 31605 | 0.028 |
| 1971 | 33752 | -0.576 |
| 1972 | 38071 | -0.405 |
| 1973 | 36910 | -0.275 |
| 1974 | 28305 | 0.156 |
| 1975 | 30740 | 0.024 |
| 1976 | 30842 | -0.338 |
| 1977 | 35528 | 0.522 |
| 1978 | 34228 | 0.172 |
| 1979 | 39283 | -0.064 |
| 1980 | 29061 | 0.996 |
| 1981 | 40195 | 0.101 |
| 1982 | 37077 | -0.121 |
| 1983 | 30598 | 0.331 |
| 1984 | 35410 | -0.205 |
| 1985 | 41899 | 0.261 |
| 1986 | 42521 | 0.074 |
| 1987 | 43558 | 0.036 |
| 1988 | 40033 | 0.738 |
| 1989 | 34792 | 0.509 |
| 1990 | 39592 | 0.167 |
| 1991 | 37193 | 0.149 |
| 1992 | 45579 | -0.813 |
| 1993 | 37605 | -0.138 |
| 1994 | 45633 | 0.226 |
| 1995 | 34882 | 0.173 |
| 1996 | 42263 | 0.158 |
| 1997 | 43422 | 0.146 |
| 1998 | 42256 | 1.087 |
| 1999 | 43723 | 0.405 |
| 2000 | 38226 | 0.043 |
| 2001 | 37609 | 0.326 |
| 2002 | 31777 | 1.085 |
| 2003 | 33099 | 0.933 |
| 2004 | 43688 | 0.196 |
| 2005 | 42976 | 0.947 |
| 2006 | 35194 | 1.321 |
| 2007 | 45971 | 0.776 |
| 2008 | 40848 | 0.113 |
| 2009 | 43534 | -0.035 |
| 2010 | 45118 | 0.526 |
| 2011 | 33888 | 1.107 |
| 2012 | 31131 | 1.349 |
| 2013 | 37396 | 0.563 |
| 2014 | 42417 | -0.085 |
| 2015 | 47709 | 0.4 |
| 2016 | 48912 | 1.105 |
| 2017 | 45031 | 1.29 |
| 2018 | 45266 | 1.197 |
| 2019 | 45845 | 1.083 |
| 2020 | 45946 | 1.055 |
| 2021 | 43309 | 0.923 |
| 2022 | 25792 | 1.358 |

**Table S2.** List of primers used in this study

| Description | Gene ID | Forward | Reverse |
| --- | --- | --- | --- |
| Drought marker | Sobic.001G405500 | CCAGCCTGAACGTGTGATAAT | CTCATAGGCAGTGGTGTTCTTC |
| Drought marker | Sobic.003G081900 | CGGATCTCCGGTTTCAGTTT | CTGCTAGGAAGCACCATGAA |
| Drought marker | Sobic.009G116700 | CGTGTGAAAGCCGTACTTAATC | CAAGTCACACCACACAACAAA |
| Heat marker | Sobic.001G426000 | GGTGAAGTCCATTCAGATCTCC | CTGCCGTTTCCTCCAATCA |
| Heat marker | Sobic.004G321000 | GATCGCCGTCTTGAAATTTGG | CTGGTGTAGGCATAACTGAGTC |
| Heat marker | Sobic.005G122500 | CGTCACATTCACAGAACAACTC | AGGTGTAGTAGTAGTCGTAGGG |
| Salinity marker | Sobic.001G378300 | GTTGTTGCTGGGTACAATGATG | ACTGCCCGAACAAGTTATAGG |
| Salinity marker | Sobic.003G292400 | GTACGGGCATTGCTGAACTA | GGTCACAATCAGTCCCTTTGA |
| Salinity marker | Sobic.003G323500 | AACCGTCAGCTTGCTCTATC | GCACTCTGCTAACCACCTAAA |
| SbEXPA11 | Sobic.004G121900 | CGACACGGATGGACAAACTAT | AGCTCTCATCGTCACTTGAAC |
| SbXTH25 | Sobic.007G086400 | CCATCATATGGAACCCGCATA | TACAGTGGGAAGGGAGTATCA |
| Internal control | Sobic.001G405500 | ACGAACTTGTTGCGGCAGAAG | GAACAAGAAGGGATGCGCTGG |
